# Inequalities in geographical access to emergency obstetric and newborn care

**DOI:** 10.2471/BLT.24.292287

**Published:** 2024-10-02

**Authors:** Aduragbemi Banke-Thomas, Lenka Beňová, Nicolas Ray, Kerry LM Wong, Charlotte Stanton, Shravya Shetty, Bosede B Afolabi

**Affiliations:** a Faculty of Epidemiology and Population Health, London School of Hygiene & Tropical Medicine, Keppel St, London WC1E 7HT, England.; b Department of Public Health, Institute of Tropical Medicine, Antwerp, Belgium.; c Institute of Global Health, University of Geneva, Geneva, Switzerland.; d Google LLC, Mountain View, United States of America.; e Department of Obstetrics and Gynaecology, College of Medicine of the University of Lagos, Lagos, Nigeria.

In 2020, an estimated 287 000 women died due to complications of pregnancy and childbirth, while 1.9 million stillbirths occurred in 2021. As many as half of maternal deaths and three in four stillbirths are preventable if women can access timely emergency care that is provided by skilled health personnel.^1^ To date, efforts of the global community to reduce maternal mortality and stillbirths have mostly focused on ensuring the availability of emergency obstetric and newborn care, minimizing financial barriers to care and, more recently, improving care quality. However, governments have given less attention to geographical accessibility and inequalities in access between and within populations. Pregnant women in low- and middle-income countries often need to seek care on their own, even in emergencies, and many face immense challenges in reaching emergency obstetric and newborn care facilities.^2^

Here we examine the geographical accessibility to emergency obstetric and newborn care in low- and middle-income settings. We argue for the use of emerging scientific evidence and contextual understanding to better identify priority problem areas, select appropriate methods, and develop solutions and targets related to assessing geographical accessibility for emergency obstetric and newborn care.

## New thinking about the problem

Inequalities in geographical accessibility to emergency obstetric and newborn care have long been reported in low- and middle-income countries. However, specific challenges vary between urban and rural settings. In rural areas, poor geographical accessibility is often attributed to long travel distances to better-equipped urban facilities and to seasonal isolation due to flooding. In urban settings, challenges mostly relate to traffic congestion, poor road conditions and sprawling slums.^3^^,^^4^ Nonetheless, interventions have mostly focused on rural rather than urban settings,^5^ although evidence suggests that the so-called urban advantage is diminishing. Indeed, peri-urban areas now report higher odds of poor pregnancy outcomes compared to rural areas.^6^^,^^7^ Rapid urbanization is contributing to this shift in outcomes. Within some urban settings in low- and middle-income countries, the urban poor and those who live in informal settlements experience disproportionate inequalities to reach emergency obstetric and newborn care compared to other urban dwellers.^8^^,^^9^ We argue that while global efforts to address inequalities in geographical accessibility in rural settings must be sustained, tackling inequalities in urban areas needs more attention.

## New thinking around the methods

Researchers have used several approaches to characterize geographical accessibility to emergency obstetric and newborn care, including those estimating distance or travel time to health facilities. However, as these approaches do not map the actual pathway and travel conditions, they fail to capture women’s lived experiences. This limitation is particularly important in urban settings because of contextual factors such as traffic congestion and the existence of large informal settlements.^10^ These factors result in high variability in travel times, and require different methods to accurately characterize inequalities in geographical accessibility. Local stakeholders participating in travel scenario workshops to establish realistic travel speeds for time estimation have described difficulties in imagining, defining and agreeing on travel speeds.^11^ Many studies estimate travel time only to the nearest health facility, despite evidence showing that many women bypass the nearest facility – even in emergencies – for various reasons, including cost of care and perception of care quality.^4^ Other women may need a referral because the first facility cannot provide the required intervention.^2^ Many studies assessing geographical accessibility rely on static, one-time assessments of travel time and facility functionality, both of which are typically dynamic in nature. These limitations reduce the usefulness of such assessments for evidence-based policy-making.

Consequently, researchers have shown increasing interest in how using data from navigation applications such as Google Maps can inform our understanding of travel time and geographical access to emergency care in low- and middle-income countries. Such data provide closer-to-reality estimates of travel time to care compared to traditional modelling methods, especially in urban areas.^10^ These applications provide opportunities to conduct revealed assessments of geographical accessibility using data from actual journeys women have taken to seek care, and potential assessments based on projected journeys a woman might take to seek care. The revealed accessibility assessment method reflects women’s lived experiences.^8^ In contrast, the potential accessibility assessment allows the incorporation of an element of choice by mapping journeys to the first, second and third nearest public and private facilities and various modes of transport, and captures journeys during peak and non-peak traffic hours.^12^ The potential accessibility assessment also allows the incorporation of other considerations needed for assessing accessibility, including cost, perception of care quality, availability of bed space and referral capacity. Together, estimates from both methods can be transformative in assessing geographical accessibility to emergency obstetric and newborn care and other health-care interventions.

## New thinking about the solutions

Efforts to address geographical accessibility to emergency obstetric and newborn care in low- and middle-income countries have included construction and upgrading of infrastructure, redesigning referral networks, providing (emergency) transportation and establishing maternity waiting homes. Most efforts to provide emergency transportation have been led by nongovernmental organizations, implemented on a small scale and not sustained.^5^ Some state-run initiatives started from nongovernmental organization-led projects focused on optimizing transportation, communication and community awareness.^5^ Regarding infrastructure, researchers proposed a health system redesign that shifts all deliveries to comprehensive emergency obstetric and newborn care facilties.^3^ However, others argue that although building new hospitals is politically attractive, it could have a negative effect on foundational primary care.^13^ In efforts to redesign health-care networks, the United Nations Population Fund has supported governments in optimizing their national facility networks to ensure good geographical access within one- or two-hour travel time thresholds in 15 countries from the World Health Organization (WHO) Regions of Africa and South-East Asia.^14^

We argue that addressing issues of geographical accessibility requires tailored approaches, as solutions that work in rural areas differ from those needed in urban settings. Addressing identified inequities calls for context-specific interventions, with the underlying principle being to bring emergency obstetric and newborn care closer to pregnant women in rural areas, and to facilitate access of pregnant women to care in urban areas. In rural settings, efforts should focus on implementing interventions such as maternity waiting homes, upgrading peripheral facilities and supporting community-based first responders. In urban areas, the focus should be on improving emergency transport and referral systems; providing legal permission for commuters in health emergencies to use bus-only lanes; and raising community awareness to give way to emergency vehicles.

## New thinking around the targets

Targets to track progress must be informed by core scientific evidence and aligned with survivability outcomes after geographical accessibility has been addressed. In 2009, when WHO published *Monitoring emergency obstetric care: a handbook*, it was considered a reasonable standard for emergency obstetric and newborn care facilities to be accessible within 2–3 hours of travel for most women. This standard was based on the estimate that two hours is the average interval between the onset of major obstetric complications and death in the absence of medical interventions. More recently, a measurable target was defined in WHO’s *Ending preventable maternal mortality (EPMM)*. The target focuses on travel time, but it is difficult to isolate the time women spend on taking decisions or establishing their capacity to seek care, making stopovers on the way to care, or waiting at other health facilities that could not provide definitive care needed from total travel time. The targets are also about most women and not all, which is inconsistent with the “leaving no one behind” motto.

Evidence from Lagos, Nigeria, shows that pregnant women who are referred and travel more than 30 minutes to receive care, and babies who are transported as little as 10 minutes directly to care have significantly higher odds of death than those who travel for shorter times than these thresholds.^6^^,^^7^ In a study conducted in Sierra Leone, travel time below 30 minutes had the best perinatal outcomes.^15^ The two-hour threshold therefore appears overly generous for urban-dwelling women in need of emergency obstetric and newborn care, and even more so for their more physiologically fragile babies. Indeed, some countries have used lower thresholds, such as one hour.^14^ However, even the two-hour threshold is difficult to attain in many sparsely populated low- and middle-income settings. Setting clinically significant thresholds focused on access to specific services should be considered.

## Going forward

While countries have made progress in optimizing geographical accessibility, we argue that new thinking around this issue is needed. Geographical accessibility assessments need to better reflect realities of travel in different settings. While traditional modelling approaches may suffice in rural areas with minimal variation in travel time, navigation-enabled mobile applications should be used for urban settings to capture access variability. Strategic partnerships are necessary to ensure availability of data for these assessments, which will also require regular, government-led updates on facility functionality. We call for adopting new real-world travel time estimates combined with geospatial databases on health-seeking behaviour, care experiences and outcomes, to inform evidence-based decision-making for health system redesign. This approach will ensure cost-effective solutions, address inequities and contribute to the realization of universal health coverage. This approach will also inform context-specific and evidence-based benchmarks for geographical accessibility that reflect real-world conditions and are clinically relevant, thereby improving maternal and newborn outcomes.

## References

[1] Paxton A, Maine D, Freedman L, Fry D, Lobis S. The evidence for emergency obstetric care. Int J Gynaecol Obstet. 2005 Feb;88(2):181–93. 10.1016/j.ijgo.2004.11.026 15694106

[2] Ameyaw EK, Njue C, Tran NT, Dawson A. Quality and women’s satisfaction with maternal referral practices in sub-Saharan African low and lower-middle income countries: a systematic review. BMC Pregnancy Childbirth. 2020 Nov 11;20(1):682. 10.1186/s12884-020-03339-3 33176732 PMC7656726

[3] Roder-DeWan S, Nimako K, Twum-Danso NAY, Amatya A, Langer A, Kruk M. Health system redesign for maternal and newborn survival: rethinking care models to close the global equity gap. BMJ Glob Health. 2020 Oct;5(10):e002539. 10.1136/bmjgh-2020-002539 33055093 PMC7559116

[4] Banke-Thomas A, Balogun M, Wright O, Ajayi B, Abejirinde IOO, Olaniran A, et al. Reaching health facilities in situations of emergency: qualitative study capturing experiences of pregnant women in Africa’s largest megacity. Reprod Health. 2020 Sep 25;17(1):145. 10.1186/s12978-020-00996-7 32977812 PMC7519554

[5] Alaofe H, Lott B, Kimaru L, Okusanya B, Okechukwu A, Chebet J, et al. Emergency transportation interventions for reducing adverse pregnancy outcomes in low-and middle-income countries: a systematic review. Ann Glob Health. 2020 Nov 18;86(1):147. 10.5334/aogh.2934 33262936 PMC7678559

[6] Banke-Thomas A, Avoka CK, Gwacham-Anisiobi U, Omololu O, Balogun M, Wright K, et al. Travel of pregnant women in emergency situations to hospital and maternal mortality in Lagos, Nigeria: a retrospective cohort study. BMJ Glob Health. 2022 Apr;7(4):e008604. 10.1136/bmjgh-2022-008604 35487675 PMC9058694

[7] Banke-Thomas A, Avoka CK, Gwacham-Anisiobi U, Benova L. Influence of travel time and distance to the hospital of care on stillbirths: a retrospective facility-based cross-sectional study in Lagos, Nigeria. BMJ Glob Health. 2021 Oct;6(10):e007052. 10.1136/bmjgh-2021-007052 34615663 PMC8496383

[8] Banke-Thomas A, Wong KLM, Collins L, Olaniran A, Balogun M, Wright O, et al. An assessment of geographical access and factors influencing travel time to emergency obstetric care in the urban state of Lagos, Nigeria. Health Policy Plan. 2021 Oct 12;36(9):1384–96. 10.1093/heapol/czab099 34424314 PMC8505861

[9] Ahmed S, Adams AM, Islam R, Hasan SM, Panciera R. Impact of traffic variability on geographic accessibility to 24/7 emergency healthcare for the urban poor: a GIS study in Dhaka, Bangladesh. PLoS One. 2019 Sep 16;14(9):e0222488. 10.1371/journal.pone.0222488 31525226 PMC6746363

[10] Banke-Thomas A, Wong KLM, Ayomoh FI, Giwa-Ayedun RO, Benova L. “In cities, it’s not far, but it takes long”: comparing estimated and replicated travel times to reach life-saving obstetric care in Lagos, Nigeria. BMJ Glob Health. 2021 Jan;6(1):e004318. 10.1136/bmjgh-2020-004318 33495286 PMC7839900

[11] Molenaar L, Hierink F, Brun M, Monet JP, Ray N. Travel scenario workshops for geographical accessibility modeling of health services: a transdisciplinary evaluation study. Front Public Health. 2023 Jan 18;10:1051522. 10.3389/fpubh.2022.1051522 36743157 PMC9889992

[12] Banke-Thomas A, Wong KLM, Olubodun T, Macharia PM, Sundararajan N, Shah Y, et al. Geographical accessibility to functional emergency obstetric care facilities in urban Nigeria using closer-to-reality travel time estimates: a population-based spatial analysis. Lancet Glob Health. 2024 May;12(5):e848–58. 10.1016/S2214-109X(24)00045-7 38614632

[13] Chabrol F, Albert L, Ridde V. 40 years after Alma-Ata, is building new hospitals in low-income and lower-middle-income countries beneficial? BMJ Glob Health. 2019 Apr 22;3 Suppl 3:e001293. 10.1136/bmjgh-2018-001293 31168419 PMC6518371

[14] Brun M, Monet J, Moreira I, Agbigbi Y, Lysias J, Schaaf M, et al. Implementation manual for developing a national network of maternity units. Improving emergency obstetric and newborn care (EmONC). New York: United Nations Population Fund; 2020. Available from: https://www.unfpa.org/sites/default/files/pub-pdf/2023%20EN_EmONC_web.pdf [cited 2024 Sep 30].

[15] van Duinen AJ, Adde HA, Fredin O, Holmer H, Hagander L, Koroma AP, et al. Travel time and perinatal mortality after emergency caesarean sections: an evaluation of the 2-hour proximity indicator in Sierra Leone. BMJ Glob Health. 2020 Dec;5(12):e003943. 10.1136/bmjgh-2020-003943 33355267 PMC7754652

